# Exploring the Role of *Staphylococcus Aureus* Toxins in Atopic Dermatitis

**DOI:** 10.3390/toxins11060321

**Published:** 2019-06-05

**Authors:** Fabio Seiti Yamada Yoshikawa, Josenilson Feitosa de Lima, Maria Notomi Sato, Yasmin Álefe Leuzzi Ramos, Valeria Aoki, Raquel Leao Orfali

**Affiliations:** Laboratory of Dermatology and Immunodeficiencies (LIM-56), Department of Dermatology, University of Sao Paulo Medical School, Sao Paulo-SP 01246-903, Brazil; faseiti@gmail.com (F.S.Y.Y.); jnilsonflima@yahoo.com.br (J.F.d.L.); marisato@usp.br (M.N.S.); yasmimleuzzi@gmail.com (Y.Á.L.R.); valeria.aoki@gmail.com (V.A.)

**Keywords:** *Staphylococcus aureus*, enterotoxins, atopic dermatitis, innate immunity, adaptive immunity, microbiome

## Abstract

Atopic dermatitis (AD) is a chronic and inflammatory skin disease with intense pruritus and xerosis. AD pathogenesis is multifactorial, involving genetic, environmental, and immunological factors, including the participation of *Staphylococcus aureus*. This bacterium colonizes up to 30–100% of AD skin and its virulence factors are responsible for its pathogenicity and antimicrobial survival. This is a concise review of *S. aureus* superantigen-activated signaling pathways, highlighting their involvement in AD pathogenesis, with an emphasis on skin barrier disruption, innate and adaptive immunity dysfunction, and microbiome alterations. A better understanding of the combined mechanisms of AD pathogenesis may enhance the development of future targeted therapies for this complex disease.

## Introduction

Atopic dermatitis (AD) is a prevalent, chronic, inflammatory, and immune-mediated skin disease [1]. Complex interactions among susceptibility genes encoding skin barrier molecules [2], inflammatory response elements, environmental factors, and infectious agents (especially *Staphylococcus aureus* and herpes virus) that lead to dysbiosis of the microbial community resident in AD skin [3,4], together with the altered immunologic status of the host, are crucial elements in the pathophysiology of AD [5,6]. Bacterial infection is a powerful trigger for AD flares and has become a matter of concern due to the widespread occurrence of antibiotic-resistant strains (methicillin-resistant *S. aureus*; MRSA) [7,8].

*S. aureus* is a Gram-positive bacterium present in 20–30% of healthy subjects. Rates of *S. aureus* carriage in AD skin reach 30–100% [9,10]. *S. aureus* produces many virulence factors which determine its pathogenicity and antimicrobial survival, including secreted toxins, enzymes, and cell-surface-associated antigens [11]. Together, these factors allow this bacterium to elude the host’s natural defenses. 

**Figure 1:**
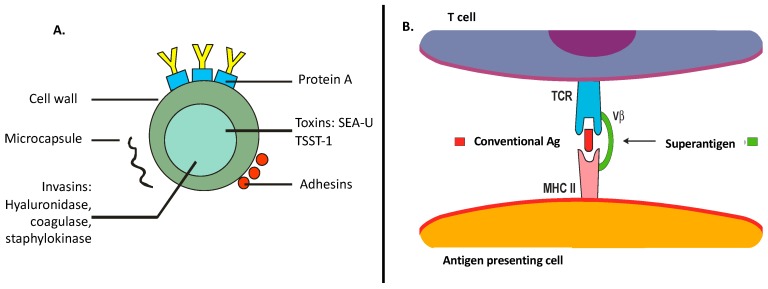
(**A**) *Staphylococcus aureus* main compounds that contribute to enhanced bacterial virulence factor. SE—staphylococcal enterotoxins; TSST-1—toxic shock syndrome toxin-1. (**B**) *S. aureus* as a superantigen: direct binding to the major histocompatibility complex (MHC) class II molecule, with specificity for the T-cell receptor (TCR)-Vβ chain.

Single-lipid membranes, surrounded by a peptidoglycan and lipoteichoic acid layer anchored by diacylglycerol, are the components of *S*. *aureus*’ cell wall [11]. Of note, *S. aureus* products include a myriad of components that play specific roles in the inflammatory/immune response, as follows: (a) Superantigens (e.g., staphylococcal enterotoxins (SE)A–U and toxic shock syndrome toxin (TSST)-1)—proteins with high mitogenic properties, leading to T- and B-cell expansions causing clonal deletion and massive cytokine production. (b) Cytotoxins (e.g., α-toxin and leukocidins)—trigger cytokine production, hemolysis, and leukocyte cell death within targeting specific cell surface receptors. (c) Enzymes (e.g., β-toxin)—trigger cytotoxicity resulting in cell death, inflammation, and tissue barrier disruptions. (d) Adhesins—cell wall receptors for epidermal and dermal laminin and fibronectin. (e) Other enzymes (e.g., proteases and/or nucleases)—mediate host protein degradations that can also act on self-proteins to degrade biofilms for bacterial dissemination. Altogether, these toxins and enzymes provide critical nutrients that are essential for bacterial growth and survival, targeting different aspects of the host’s immune response and therefore contributing to *S. aureus* virulence (Figure 1A) [9,11,12].

*S. aureus* may act as a conventional antigen or as a superantigen to activate T cells. Unlike conventional antigens, superantigens do not need to be processed and can bind directly to the surface of the major histocompatibility complex (MHC) class II molecule with T-cell receptor (TCR) Vβ-chain specificity, generating a polyclonal T-cell stimulation (Figure 1B) [9,13,14]. Our group previously studied the effects of distinct antigens and mitogens (tetanic toxoid, *Candida albicans*, SEA, and phytohemagglutinin) in the proliferative response of peripheral blood mononuclear cells (PBMCs) from adults with AD and identified a decreased PBMC proliferation response to these stimuli, suggesting a compromised immune profile due to staphylococcal chronic skin colonization [13]. In this review, we focus on the role/mechanisms of *S. aureus* in the pathogenesis of AD. 

## 1. Role of *S. Aureus* Toxins in the Disruption of the Skin Barrier and Innate Immunity

The skin is the first barrier to pathogens and, as a frontline defender, shows a remarkable immune function due to the presence of a diverse set of cells such as keratinocytes, dendritic cell (DCs), mast cells (MCs), and lymphocytes [15]. *S. aureus* has higher adhesion to corneocytes from AD patients than to those in non-AD individuals. This enhanced binding is mediated by the interaction between bacterial adhesins, such as protein A, and host surface receptors [16]. Moreover, staphylococcal toxins do have immune stimulatory abilities, and there is higher production of such toxins in strains isolated from AD individuals [17].

Most of the innate immune response relies on the identification of nonself molecules by pattern recognition receptors (PRRs). Toll-like receptors (TLRs) are one of the most critical classes of PRRs, particularly bacterial motifs. As membrane-associated receptors, they are expressed at the cell surface where they can survey the extracellular environment for pathogen clues, but are also found in endosomes, thus tracking intracellular stimuli [18]. TLR2 has been identified as a key receptor for *S. aureus* recognition due to its ability to identify lipoproteins, which are abundantly expressed in the cell wall of Gram-positive bacteria [18]. Interestingly, keratinocytes from AD patients show a reduced response to TLR2 agonists, with lower production of IL-6, IL-8, CCL20, and matrix metalloproteinase-9 (MMP-9) [19]. Although the mechanisms involved in this diminished response are still unknown, TLR2 expression in these cells are equivalent in both AD and healthy subjects. The dysfunction in TLR response in AD patients can be associated with deficiencies in the signaling components. Some studies point out that alterations in the myeloid differentiation protein (MyD88) pathway is needed for the development of SEB-induced AD-like phenotype [20,21].

In parallel with TLRs, another remarkable class of PRRs is the nucleotide-binding oligomerization domain (NOD)-like receptor (NLR) family. NLRs mainly work by forming multicomponent platforms known as inflammasomes, which drive the activation of protease caspase-1; *S. aureus* components activate the NLRP3 inflammasome [22]. In AD, *S. aureus* α-hemolysin is capable of activating this complex in keratinocytes, and inflammasome activation is greatly compromised due to the reduced expression of its components, driven by the action of T helper (Th)2 cytokines [23]. Nonetheless, keratinocytes may circumvent this limitation by producing IL-1β and IL-18 in an inflammasome-independent fashion in response to other *S. aureus* toxins, such as phenol-soluble modulins (PSM), which directly cause cell lysis and cytokine extravasation [24].

Moreover, α-hemolysin contributes to disruption of the skin barrier by interacting with lipid sphingomyelin, generating pores in the cell membrane that culminate in keratinocyte lysis. AD patients show a higher susceptibility to α-hemolysin action once Th2 cytokines promote the downregulation of acid sphingomyelinase, which increases the availability of α-hemolysin targets [25]. In addition, AD patients show defective filaggrin expression, contributing to disease severity. Filaggrin is highly expressed in differentiated keratinocytes and promotes the secretion of acid sphingomyelinase and resistance to α-hemolysin activity [26,27].

The α-toxin can also compromise the keratinocyte layer by altering E-cadherin integrity. Curiously, this effect can be blocked by pharmacological activation of the G-protein-coupled estrogen receptor (GPER), which ultimately promotes repression of the host protein ADAM10, a receptor for α-hemolysin responsible for cleaving E-cadherin [28]. Moreover, *S. aureus* could compromise the keratinocyte biology at the transcriptional level. Brauweiler et al. (2017) showed that *S. aureus* lipoteichoic acid (LTA) profoundly affects the keratinocyte genetic program, repressing genes associated with cell differentiation by interfering with the protein p63, which is a master transcription regulator in skin [29].

Using a murine model of *S. aureus* epicutaneous exposure to induce an AD-like disease, Liu et al. (2017) uncovered the role of PSMα in promoting IL-36 secretion by keratinocytes, which directly activates T cells to produce IL-17 and drives skin inflammation [30]. Interestingly, Baldry et al. (2018), using the same approach, suggested that another PSM, such as δ-toxin, would be more relevant to induce inflammation once the use of a mutant strain for δ-toxin or the pharmacological inhibition of its production with solonamide could efficiently block AD development [31]. In support of the latter findings, Matsuo et al. (2018) showed that δ-toxin, when combined with ovalbumin, could promote an AD-like phenotype without any other bacterial component, inducing production of the Th2-recruiting chemokines CCL17 and CCL22 in keratinocytes [32].

In sharp contrast to the epicutaneous model, previous systems such as tape stripping, in combination with chemically induced AD-like lesions, did not indicate a remarkable contribution of *S. aureus* toxins in AD pathogenesis. It is possible that the effects of toxins such as SEA were mostly coadjuvant, with mild contributions to the cytokine pool and disease phenotype due to the harsh stress caused by this method [33]. However, evidence from human skin biopsies did show a high expression of SEA in AD tissues, which suggests an indisputable contribution of these toxins to immune activation [34].

In addition to keratinocytes, *S. aureus* toxins also interfere in the activity of immune cells, particularly those which reside in or are recruited to the skin. Saloga et al. (1996) investigated the immune response kinetics after a single intracutaneous dose of SEB and showed the activation of resident Langerhans cells, degranulation of MCs, and recruitment of eosinophils followed by the influx of mononuclear cells (monocytes/macrophages and lymphocytes) [35]. Thus, staphylococcal toxins alone may exert multiple actions on skin immunity.

A milestone work from Nakamura et al. (2013) demonstrated that the δ-toxin is one of the main activators of MCs, triggering their degranulation and reinforcing the causal link between *S. aureus* colonization and AD development [31]. δ-Toxin promotes MC degranulation by activating the receptor Mas-related G-protein-coupled receptor member X2 (MRGPRX2), promoting Ca^2+^ influx and release of granule content. As MRGPRX2 is also expressed in keratinocytes, this suggests that the contribution of δ-toxin to AD pathogenesis can be extended to other cell types [36]. Interestingly, SEB was initially proposed to inhibit IL-4 production in the MC line HMC-1 [37], but whether this effect applies to primary MCs is still unknown.

Other granulocytes can also infiltrate AD lesions. Basophils can release histamine in response to a diverse set of *S. aureus* toxins in an IgE-dependent manner [38,39], while eosinophils respond to SEB, protein A, and peptidoglycan through the receptor CD48 with secretion of the enzyme eosinophil peroxidase and the neutrophil chemoattractant IL-8 [40]. Staphylococcal toxins inhibit eosinophil apoptosis, therefore not only triggering but also perpetuating the allergic response by postponing cell death [41]. 

DCs belongs to a key immune population in the initiation and polarization of T-cell responses due to their ability to present antigens and to secrete polarizing factors according to different stimuli [42,43]. Concerning their role in AD pathogenesis, SEB-stimulated DCs develop a Th2-polarizing phenotype, which supports the atopic profile in these patients [44]. In turn, Th2 products may affect the innate response as described by Kasraie et al. (2016), who showed that IL-31, a T-cell cytokine involved in pruritus, activates monocytes and macrophages, enhancing the inflammatory response [45].

Curiously, the altered DC phenotype in AD patients is not limited to resident skin populations. Kapitány et al. (2017) observed that CD1c^+^ blood pre-DCs, precursors of dermal DCs from AD patients, have an altered response (either constitutively or after SEB stimulation), with heightened production of Th2-associated chemokines [46]. These authors also noticed that these cells are more premature, which could be the result of the particular serum cytokine signature in those patients or due to an altered hemopoiesis with early release of immature cells. These results reinforce the notion that AD is far beyond a restricted dermatological disease but rather a condition of systemic immune dysfunction. Indeed, some works showed that epicutaneous SEB sensitization can enhance Th17-driven lung inflammation by inducing the production of the IL-17 polarizing cytokine IL-6 [47] and that α-toxin can help infection by viruses, such as herpes simplex virus 1, by facilitating viral entry into the host cell [48].

Intriguingly, while SEB induced apoptosis of the human monocytic cell line THP-1 [49], similar to what was described for T cells [50], TSST-1 showed the opposite effect, protecting primary monocytes from death [51]. The proposed mechanism of action for SEB-induced death relies on induction of TNF-α, which activates the extrinsic apoptotic pathway [49], while TSST-1 promotes GM-CSF (Granulocyte Macrophage Colony-Stimulating Factor) secretion, promoting cell survival [51]. The scenario that prevails in the host may depend on multiple factors, such as the host immune response or differences in the toxin profile secreted among different *S. aureus* strains.

SEB efficiently activates monocytes, inducing production of TNF-α in a TLR2- and TLR4-dependent way, in nonatopic individuals compared to AD patients, corroborating the anergic/exhausted profile described in AD [52]. Similarly, α-toxin was a strong inducer of CXCL10 in macrophages from healthy individuals but not in AD counterparts [53], which could contribute to a dampened Th1 response in atopic subjects. A possible explanation could rely on the reduced expression of immune receptors such as the lower expression of TLR2 in AD macrophages [54], which can compromise the triggering of an effective immune response, allowing bacterial overgrowth. Although α-toxin was shown to promote TLR2 expression in monocytes from non-AD subjects, thus enhancing their cytokine response [55], the effects on AD cells can have a divergent outcome, supporting the atopic response.

The host genetic background must be considered for a full appreciation of the immune mechanisms triggered by staphylococcal toxins. Krogman et al. (2017), using transgenic mice expressing different alleles of HLA-DR (Human Leukocyte Antigen – DR isotype) molecules, showed how the immune response changes in response to TSST-1, with some alleles favoring an increased inflammatory response and tissue damage [56]. Nieburh et al. (2008) identified a single-nucleotide polymorphism in the TLR2 gene from AD patients that increases the production of IL-6 and IL-12p70 by monocytes [57], potentially aggravating their inflammatory reaction. Those genetic differences may help to explain why AD patients show very intense *S. aureus* proliferation and respond with deleterious inflammation, even though the bacterium is a component of skin microbiota. Another variable that should be considered is the age of the host, since atopic children may exhibit different response kinetics to bacterial antigens than adults [52].

Newer evidence suggests that in addition to their inflammation-promoting roles, staphylococcal toxins may also counterbalance the host regulatory mechanisms. Though glucocorticoids (GC) are a common therapeutic strategy in AD management, utilizing the glucocorticoid receptor (GR) to dampen the inflammatory reaction, some patients become unresponsive to treatment. Huang et al. (2018) showed that SEB can block GC action by impeding the nuclear translocation of GR in keratinocytes, favoring the persistence of inflammation [58]. Also, staphylococcal enterotoxins can modulate the induction of myeloid-derived suppressor cells (MDSCs). While lower doses of toxins favor MDSC generation, higher doses (the likely scenario in AD patients who show *S. aureus* overgrowth) modulate MDSC response, thus circumventing inflammation control [59].

## 2. *S. Aureus*: Straight Relationship with Adaptive Immunity in AD

Decreased skin microbiota diversity associated with high susceptibility to *S. aureus* colonization [10,60,61] is described in the skin of AD patients. *S. aureus* is able to secrete more than 20 different toxins that play an important role in AD adaptive immune response. Around 60% of isolated *S. aureus* strains are capable of secreting exotoxins [62], acting on distinct T- and B-cell pathways. As superantigens, they can stimulate cytokine secretion and T-cell proliferation; as conventional allergens, they can induce production of staphylococcal-specific IgE exotoxin [63]. 

Classically, AD immune pathogenesis is described as a dysfunction of the Th1/Th2 balance [64]. Acute flares of AD are characterized by Th2 and Th22 cell infiltrates, whereas Th1 cells are detected in chronic lesions [65]. However, there are reports of novel subsets of human Th cells, such as Th17 and Th22 cells [66], that are present in the inflammatory response of AD [5,27,64,67]. AD has several immune subtype profiles, all having a common Th2/Th22 polarization, but also displays differential immune skewing, such as increased Th17 that has been identified in the skin of intrinsic, Asian, and early pediatric AD patients [68]. Th17-derived IL-17 is able to coordinate local tissue inflammation through upregulation of proinflammatory cytokines and chemokines, including IL-6, TNF-α, IL-1β, CXCL1, CCL2, CXCL2, CCL7, and CCL20 [67]. In cooperation with IL-17, IL-22 triggers antimicrobial peptide production and initiates an acute phase response [69,70]. In recent publications, there was expression of Th22 cells and IL-22-producing CD8^+^ T cells present in acute and chronic AD skin lesions [65,71].

The major Th2 cytokine involved in AD acute flares is IL-4, which mediates enhanced expression of fibronectin and fibrinogen, working as adhesion molecules for *S. aureus* and therefore contributing to AD skin bacterial chronic colonization. AD patients with colonized skin by *S. aureus* have increased disease severity, elevated eosinophil blood counts, total IgE serum levels, CCL17 and periostin (Th2 biomarkers) plasma levels, and suppressed activity of Treg cells [72] when compared with those without *S. aureus* colonization [73], corroborating the chronic immune activation described in AD. 

Th2 cells can also incite B cells, which leads to IgE and IL-5 production, stimulating maturation and survival of eosinophils in some types of AD [65]. Furthermore, purified eosinophils from AD patients stimulated with TLR2/6 agonist and SEB showed decreased levels of TIMP-1, TIMP-2, and CCL5, revealing a potential breakdown in the remodeling process mediated by eosinophils [74].

B-cell activation can also be modulated by *S. aureus* exotoxins (working as allergens) and stimulating exotoxin-specific IgE antibody production. SEA- or SEB-specific IgE levels are significantly elevated in the plasma of children with AD and correlate with disease severity when compared with non-AD children [75]. Enhanced production of IgE antibodies against *S. aureus* antigens is also described in adults with AD and is associated with asthma severity [76]. B cells are also a relevant source of cytokine secretion by a T-cell-independent pathway. Parcina et al. (2013) demonstrated that protein A, an important virulence factor of *S. aureus*, induced increased B-cell proliferation and regulatory B cells (Breg) secreting IL-10 via the plasmacytoid dendritic cells (pDC) pathway [77], suggesting a tolerogenic immune profile in AD. 

Pruritus is one of the major symptoms in AD patients and it is associated with impaired quality of life, as demonstrated in studies evaluating the patients’ perspectives of the disease using PO-SCORAD [78,79]. IL-31 is a Th2 cytokine associated with AD severity and pruritus [80,81]. Staphylococcal toxins, such as α-toxin and SEB, are capable of inducing a potent secretion of IL-31 by CD4^+^ T cells in AD patients [45,82]. 

**Figure 2:**
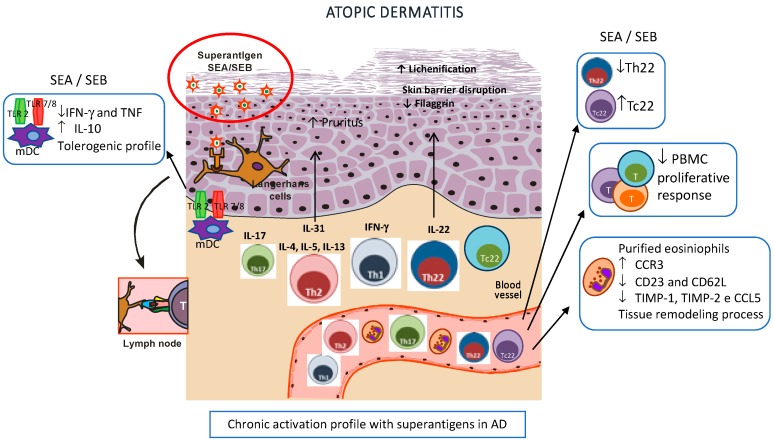
Superantigen-activated dendritic cells stimulate T helper (Th)2 cells to produce IL-4, IL-5, IL-13, and IL-31, leading to skin barrier disruption, decreased antimicrobial peptide production, impaired keratinocyte differentiation, and pruritus. In chronic atopic dermatitis (AD), there is an enrollment of Th1, Th22, and Th17 subsets that leads to epidermal thickening and abnormal keratinocyte proliferation (lichenification). Effects of staphylococcal enterotoxins in AD: 1. Dysfunctional CD4^+^ IL-22-secreting T cells and upregulated Tc22 cells. 2. Reduced peripheral blood mononuclear cell (PBMC) proliferative response corroborating an exhausted immune profile. 3. Increased frequency of CCR3^+^ and decreased expression of CD23 and CD62L receptors, and TIMP-1, TIMP-2, and CCL5 in purified eosinophils of AD patients even in a nonstimulated condition, indicating a potential breakdown in the tissue remodeling process in AD mediated by eosinophils. 4. Enhanced frequency of IL-10 under TLR4 and decreased frequency of IFN-γ and TNF under TLR2 and 7/8 stimulation in classic mDC (myeloid dendritic cells), indicating a tolerogenic profile in AD. All these findings together corroborate the chronic activated profile related to superantigens in AD pathogenesis.

In Figure 2, we have summarized the noteworthy findings in AD pathogenesis related to chronic staphylococcal enterotoxin activation described in previous studies by our group.

## 3. Microbiome and AD

An assembly of microbiota resides our human skin and cohabits in an established balance [10]. The molecular approach to the human microbiome reveals high diversity of skin microbiota within and between distinct topographical regions [10,61,83,84].

In patients with AD, the skin microbiota is altered by endogenous factors, such as skin barrier protein mutations (filaggrin, among others) or exogenous stimuli, such as soaps, topical corticosteroids, and antibiotics, leading to a modified/noneffective response of the host to allergens, pathogens, and tissue damage [85].

The microbiome can exert both beneficial and harmful influences in AD skin once it interacts with the local immune system [86]. *S. aureus* is directly correlated with increased expression of IL-4, IL-13, IL-22, IL-31, TSLP, and other cytokines and decreased expression of cathelicidin, evidencing the impact of skin dysbiosis on disease exacerbation [87]. An example of a beneficial relationship between bacteria and the skin is that the commensal bacterium *Staphylococcus epidermidis* modulates TLR3-dependent inflammation by initiating a TLR2-mediated cross-talk mechanism to suppress inflammation, indicating that more microbiome diversity seems to be more beneficial once a diverse ecosystem is more resistant [88], suggesting that the timing of exposure to commensal bacteria may affect the development of tolerance.

When analyzing lesional skin and *S. aureus* colonization in AD subjects, there is evidence of loss of microbial diversity during acute flares in patients with AD, in contrast with microbiome diversity restoration after successful anti-inflammatory treatment [61]. Kennedy et al. showed relevant findings such as the absence of *S. aureus* colonization of children prior to AD onset and colonization of antecubital fossa with commensal staphylococci at month two in infants, which is associated with decreased incidence of AD at the age of one year [89].

Furthermore, the characterization of the microbiome in children and adults with AD still requires more studies. Shi et al. [90] recently published data on microbiome studies performed in both pediatric and adult patients with AD. These authors detected significant differences in the microbiome profile of AD individuals, with two defined patterns according to age (young children and adults–teenagers). Microbiome diversity was increased in nonlesional skin of young patients when compared with adults. *Staphylococcus* was abundant in nonlesional and lesional AD skin in both age groups, suggesting susceptibility to pathogen colonization [90].

Recent studies indicate that certain strains of commensal flora composed of coagulase-negative staphylococci (CoNS) compete with *S. aureus* on the skin, increasing antimicrobial peptide production [10,91]. Additional studies in AD individuals are needed to confirm whether colonization with commensal bacteria will modulate the uncontrolled immune response with clinical improvement of the cutaneous lesions in AD patients. 

## 4. Perspectives Targeting *S. Aureus* in AD

Increasing evidence corroborates that *S. aureus* has a pivotal role in AD pathogenesis, correlating with disease flares and severity. However, many questions remain unresolved, especially regarding the best strategy against *S. aureus* in AD skin.

Well-established AD treatment options, such as narrowband UVB phototherapy, systemic cyclosporine, topical corticosteroids, and calcineurin inhibitors, indicate that therapeutic success is a reflection of the immunomodulatory mechanism of action suppressing staphylococcal-enterotoxin-activated T cells, improving the skin barrier function, and consequently decreasing *S. aureus* colonization [92]. Recent assays have evaluated the effect of dupilumab (anti-IL-4Rα) on the host–microbe interface in atopic dermatitis (available online in clinicaltrials.gov, identifier: NCT03389893) [92]. Likewise, other monoclonal antibodies are under investigation, for example, nemolizumab (CIM331), a humanized antibody against the IL-31 receptor [93] (clinicaltrials.gov, identifier: NCT01986933). Furthermore, it is important for future research to address the interactions between the gut and skin microbiomes, including *S. aureus* species, and therapies targeting the immune system via microbioma modulation

A recent AD clinical trial utilizing topical application of commensal *Roseomonas mucosa* demonstrated significant decreases in disease severity scores, topical steroid requirements, and *S. aureus* colonization, with no adverse events or treatment complications [94].

We also have to consider that vaccines against *S. aureus* represent a possible novel approach to manipulating the AD skin microbiome. Next-generation anti-*S. aureus* vaccines will require an association between targeting specific effector T-cell subsets combined with inducing specific neutralizing antitoxin antibodies [10,92,95].

One remarkable point for future therapeutic target strategies for AD should address the interaction between host and pathogen, focusing on the pathogenic role of staphylococcal enterotoxins in modulating cytokine release by activated effector T cells and affecting population-level responses to pathogens.
